# *Mu* Transposon Insertion Sites and Meiotic Recombination Events Co-Localize with Epigenetic Marks for Open Chromatin across the Maize Genome

**DOI:** 10.1371/journal.pgen.1000733

**Published:** 2009-11-20

**Authors:** Sanzhen Liu, Cheng-Ting Yeh, Tieming Ji, Kai Ying, Haiyan Wu, Ho Man Tang, Yan Fu, Dan Nettleton, Patrick S. Schnable

**Affiliations:** 1 Interdepartmental Genetics Graduate Program, Iowa State University, Ames, Iowa, United States of America; 2 Department of Genetics, Development, and Cell Biology, Iowa State University, Ames, Iowa, United States of America; 3 Center for Plant Genomics, Iowa State University, Ames, Iowa, United States of America; 4 Department of Agronomy, Iowa State University, Ames, Iowa, United States of America; 5 Bioinformatics and Computational Biology Program, Iowa State University, Ames, Iowa, United States of America; 6 Department of Statistics, Iowa State University, Ames, Iowa, United States of America; 7 Center for Carbon Capturing Crops, Iowa State University, Ames, Iowa, United States of America; Fred Hutchinson Cancer Research Center, United States of America

## Abstract

The *Mu* transposon system of maize is highly active, with each of the ∼50–100 copies transposing on average once each generation. The approximately one dozen distinct *Mu* transposons contain highly similar ∼215 bp terminal inverted repeats (TIRs) and generate 9-bp target site duplications (TSDs) upon insertion. Using a novel genome walking strategy that uses these conserved TIRs as primer binding sites, *Mu* insertion sites were amplified from *Mu* stocks and sequenced via 454 technology. 94% of ∼965,000 reads carried *Mu* TIRs, demonstrating the specificity of this strategy. Among these TIRs, 21 novel *Mu* TIRs were discovered, revealing additional complexity of the *Mu* transposon system. The distribution of >40,000 non-redundant *Mu* insertion sites was strikingly non-uniform, such that rates increased in proportion to distance from the centromere. An identified putative *Mu* transposase binding consensus site does not explain this non-uniformity. An integrated genetic map containing more than 10,000 genetic markers was constructed and aligned to the sequence of the maize reference genome. Recombination rates (cM/Mb) are also strikingly non-uniform, with rates increasing in proportion to distance from the centromere. *Mu* insertion site frequencies are strongly correlated with recombination rates. Gene density does not fully explain the chromosomal distribution of *Mu* insertion and recombination sites, because pronounced preferences for the distal portion of chromosome are still observed even after accounting for gene density. The similarity of the distributions of *Mu* insertions and meiotic recombination sites suggests that common features, such as chromatin structure, are involved in site selection for both *Mu* insertion and meiotic recombination. The finding that *Mu* insertions and meiotic recombination sites both concentrate in genomic regions marked with epigenetic marks of open chromatin provides support for the hypothesis that open chromatin enhances rates of both *Mu* insertion and meiotic recombination.

## Author Summary

Genomic insertion sites of *Mu* transposons were amplified and sequenced via next generation technology, revealing more than 40,000 non-redundant *Mu* insertion sites that are non-uniformly distributed across the maize genome and within genes. Along chromosomes, frequencies of *Mu* transposon insertions are strongly correlated with recombination rates. Although both *Mu* and recombination occur preferentially in genes, gene density does not fully explain these patterns. Instead, the finding that *Mu* insertions and meiotic recombination sites both concentrate in genomic regions marked with epigenetic marks of open chromatin provides support for the hypothesis that open chromatin enhances rates of both *Mu* insertion and meiotic recombination.

## Introduction

Gene knockouts are indispensable tools for genetic and functional genomics. The maize *Mutator* (*Mu*) transposon is the most active DNA transposon in plants [1]. In maize, a model species for which transformation can be achieved at only a low efficiency, *Mu* insertion mutagenesis has been an important tool for cloning genes due to its high copy numbers and high rate of germinal transposition [1],[2],[3]. In addition, because *Mu* elements do not exhibit a preference for transposition to nearby sites [4], as is the case for Ac/Ds transposons [5], they are ideally suited for genome-wide mutagenesis screens.

The *Mutator* transposon family is a two-component system. *MuDR* controls the transposition of itself and the other classes of the 12 nonautonomous *Mu* elements that have been reported so far [6]. All *Mu* elements share highly similar ∼215 bp terminal inverted repeats (TIRs) and upon insertion generate 9-bp target site duplications (TSDs) directly flanking *Mu* elements. *Mu* exhibits a preference for insertion in genes [7],[8],[9]. In addition, a few case studies reported a preference for insertion within 5′-UTRs or exons of genes [7],[8],[9],[10].

Although many investigations have been conducted on *Mutator* transposons, little is known about the genome-wide distribution of *Mu* insertions sites and the mechanisms by which these sites are selected. In this study, ∼965,000 *Mu* flanking sequences (MFSs) were obtained from 454 pyrosequencing libraries generated via Digestion-Ligation-Amplification [11], a novel approach for amplifying unknown sequences flanking known sequences. Analyses of these MFSs revealed 21 novel *Mu* TIR sequences and 324 genic *Mu* insertion hotspots that each contains ≥9 independent *Mu* insertions. Within genes, the *Mu* insertions exhibited a pronounced preference for 5′-ends with the strongest preference near transcription start sites. Additionally, regions close to the ends of chromosomes experience more *Mu* insertions than do peri-centromeric regions. This non-uniform pattern is similar to chromosomal distributions of recombination events and gene density. However, gene density does not fully explain the non-uniformity in genome distribution of *Mu* and recombination. Analyses using both cytosine methylation and histone modification data [12],[13] revealed a strong correlation between *Mu* insertion and cytosine methylation, H3K4me3 and H3K9ac modifications. *Mu* insertions and meiotic recombination sites both concentrate in genomic regions marked with epigenetic marks of open chromatin. We, therefore, hypothesize that open chromatin structure plays a key role in determining site selection of both *Mu* insertions and meiotic recombination events.

## Results

### The application of DLA-454 strategy in sequencing MFSs

DLA is a PCR-based method to amplify unknown sequences flanking known sequences [11]. DLA was adapted to sequence *Mu* flanking sequences using 454 pyrosequencing, a strategy that is termed DLA-454 [11]. DLA is a novel adaptor-mediated PCR-based method that uses a single-stranded oligo as an adaptor and the conserved ∼215 bp TIRs of *Mu* transposons as primer binding sites to amplify MFSs. In DLA-454, 6-bp barcodes [14] are inserted between the 454 primer A and a *Mu*-specific primer, while an adaptor primer, Nsp-P, is appended to the 454 primer B. The resulting library is sequenced using 454 primer A. By doing so, sequencing reads should begin at the barcode, followed by the *Mu*-specific primer and a portion of the TIR (pTIR), and end with the MFS or in cases of short MFSs the Nsp-P primer. From two technically replicated 454 GS-FLX runs, ∼964,808 reads were obtained. 99% of these sequences can be unambiguously categorized using the barcodes because the first 6 bp of each read exactly matched one of the barcode sequences. A two-step trimming strategy (Methods) was applied to remove barcodes, *Mu* primer, amplified *Mu* TIR, 454 primer B and the Nsp-P adaptor primer to obtain MFSs. Based on the results of this two-step trimming process, almost all (99.7%) reads include the *Mu*-specific primer and over 94% carry amplified *Mu* TIR sequences, demonstrating that most reads are generated from sites that contain a *Mu* insertion. Those trimmed MFSs (638,492) that were associated with TIR sequences were aligned to the maize B73 reference genome (B73 RefGen_v1) provided by the Maize Genome Sequencing Project (MGSP) using BLASTN (Figure S1). 58% (370,632/638,492) of the trimmed MFSs satisfied our stringent alignment cut-offs (Methods). This rate of mapping is comparable to that obtained by aligning Mo17 reads (sequenced by Joint Genome Institute using 454 pyrosequencing) to the B73 RefGen_v1 using the same criteria (data not shown). Of the aligning MFSs, 98.6% (365,600/370,632) could be uniquely mapped to a single position on the B73 RefGen_v1 and the positions of the corresponding *Mu* insertions determined. SNP identified between the MFSs and the sequences of the B73 RefGen_v1 were used to distinguish independent *Mu* insertions in different plants at the same genomic positions.

### Novel *Mu* elements

**Figure 1:**
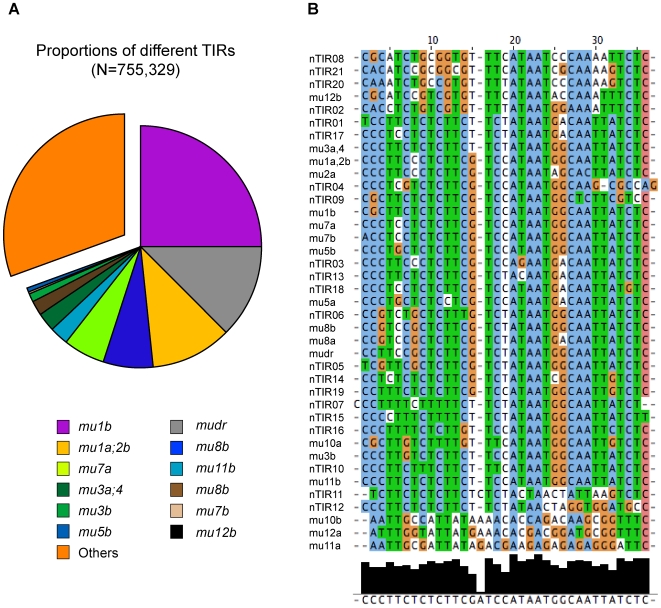
Frequencies of known and novel *Mu* pTIRs. (A) Proportions of different 34 bp pTIRs detected in the 454 dataset. The codes “a” and “b” designate arbitrarily defined left and right TIRs of a given *Mu* element. (B) Clustalw-based clustering of novel pTIRs (nTIRs), each of which was supported by at least 100 reads and exhibited a minimum edit distance of 2 from all previously described pTIRs (Methods).

About 70% (524,696/755,329) of the 454 reads that resulted from the first trimming contained 34 bp pTIR sequences that perfectly matched known pTIRs. pTIRs from all but one of the previously described *Mu* elements were detected. Assuming the frequency at which pTIRs were recovered is correlated with the frequency of the corresponding classes of *Mu* elements in the *Mu* stocks, we can conclude that *Mu1* and *MuDR* have the highest copy numbers (Figure 1A). Only a few 454 reads contained pTIRs from *Mu12* and none contained *Mu10* pTIRs. The two TIRs (left and right) of most *Mu* elements are not perfectly conserved. This allowed us to determine that TIRs from both sides of six classes of *Mu* elements (*Mu1*, *Mu3*, *Mu4*, *Mu7*, *Mu8* and *MuDR*) could be successfully amplified via DLA-454. MFS from only one side of four classes of *Mu* elements (*Mu2*, *Mu5*, *Mu11* and *Mu12*) were recovered in the DLA-454 data set (Figure 1).

Approximately, 31% of the DLA-454 reads contain pTIRs that do not perfectly match any known pTIRs. These novel sequences could be the result of sequencing errors or be evidence for the presence of novel pTIRs. We stringently required 34-bp pTIRs to have a minimum edited distance (MED) of at least 2 relative to all known pTIRs before classifying them as potentially novel pTIRs (Methods). A total of 21 novel pTIRs each of which has at least 100 supporting reads were identified (Figure 1B, Table S1). Eight of the *Mu* elements associated with these novel pTIRs were PCR amplified using the TIR primer in combination with primers designed based on the MFSs associated with the novel pTIR. Seven of the PCR products were successfully sequenced using Sanger technology. All seven novel pTIRs contained the expected polymorphisms relative to known pTIRs, suggesting that this data set has defined 21 novel *Mu* TIRs. Among the 21 novel TIRs (nTIRs), 13 were associated with multiple independent MFSs (and one, nTIR14, was associated with over 100 independent MFSs), suggesting that they are or were mobile.

### Genic hotspots for *Mu* insertions

It has previously been established that *Mu* insertions exhibit a preference for typically low-copy genes as compared to non-genes [7],[8],[9]. Our first observation in support of this preference was that only ∼6% of all trimmed MFSs (600,139/638,492) contain repeat sequences as per Emrich *et al.*, 2004 [15]. In addition, more than 98% of mappable MFSs (365,600/370,632) could be uniquely mapped to the genome even though up to 80% maize genome is repetitive [16],[17],[18]. To more directly test whether this preference of *Mu* elements to insert into genes holds true in our data set, we examined the numbers of *Mu* insertions in all of the 32,540 annotated genes in the MGSP's “filtered gene set” [18]. Even though the filtered gene set comprises only 7.5% of the genome, almost 75% of the mapped insertions are located within the 13,307 filtered genes. Similar results were obtained when these analyses were repeated with less stringently called gene sets. We therefore conclude that consistent with prior studies, *Mu* exhibits a strong preference for genic regions.

We then asked whether certain genes are “hotspots” for insertion. To do so, we used a simulation to determine that the probability of one or more genes acquiring nine or more insertions would be rare (p<0.05) if all genes were equally likely to acquire *Mu* insertions (see Methods). In the experimental data, 1% (324/32,477) of the filtered gene set had nine or more *Mu* insertions. Variation in gene length was not considered in this simulation because the correlation between *Mu* insertions and gene length is very low (r = 0.1). We used this set of genic “hotspots” to test the hypothesis that genes that experience high frequencies of *Mu* insertions are expressed at higher than average levels. Gene expression levels were estimated using mRNA-seq data from several tissues (Methods). Both hotspot genes (≥9 *Mu* insertions) and all genes that contained 1–8 *Mu* insertions have significantly higher levels of gene expression than genes without *Mu* insertions (Wilcoxon-test, all p-values<0.001, Table 1). Hotspot genes exhibit higher levels of gene expression than those genes with 1–8 *Mu* insertions (Wilcoxon-test, all p-values<0.001, Table 1). This relationship was observed consistently using data from each of three independent mRNA-seq experiments conducted using different tissues. Hence, we conclude that genes that experience elevated rates of *Mu* tend to be expressed at higher than average levels.

**Table 1:** *Mu* insertion versus gene expression.

Category	No. Genes	Mean No. reads from various mRNA-seqs		
		B73_L1^1^	B73_L2^2^	F_1__Seedling^3^
Gene	32,477	73	230	315
Zero-*Mu*-gene^4^	19,170	50	156	235
*Mu*-gene^5^	12,983	105*	335*	428*
Hotspot gene^6^	324	133* ^,^ **	423* ^,^ **	542* ^,^ **

1 Solexa mRNA-seq of L1 layer of shoot apical meristem (SAM).

2 Solexa mRNA-seq of L2 layer of shoot apical meristem (SAM).

3 Solexa mRNA-seq of 14-day seedlings from reciprocal F_1_ (B73×Mo17 and Mo17×B73). Data from reciprocal crosses were pooled for this analysis.

4 Genes without *Mu* insertions.

5 Genes with 1–8 *Mu* insertions.

6 ≥9 *Mu* insertions per gene.

*: p-value<0.001, Wilcoxon-test of gene expression with zero-*Mu*-gene group.

**: p-value<0.001, Wilcoxon-test of gene expression with *Mu*-genes.

### *Mu* insertions exhibit a preference for the 5′-ends of genes

Previously, several studies identified a tendency for *Mu* insertions to target the 5′ ends of genes. For example, Hardeman and Chandler 1989 [19] reported a preference for the first two exons of *bronze1* gene and Dietrich *et al.* 2002 [10] reported a pronounced preference for the 5′ UTR of the *glossy8* gene. This pattern has been confirmed later in multiple genes [12],[20]. To explore the distribution of *Mu* insertions within genes in our data set we used a set of full-length cDNAs [21] to generate a set of genes (N = 15,050) whose complete structures could be defined (the “flcDNA gene set”; Methods). After aligning the MFSs to the flcDNA gene set, the average numbers of *Mu* insertions per Mb were computed for each genic region (e.g., 5′ and 3′ UTRs, exons and introns) across all genes in the flcDNA gene set. A Pearson's Chi-square test (see legend of Figure S2) supported the hypothesis that frequencies of *Mu* insertions vary significantly across genic regions (*χ* ^2^ = 16,375, df = 7, p-value<2.2e-16). Overall, the 5′ most exons of genes had the highest frequencies of *Mu* insertions per Mb, particularly the 5′ UTRs and regions further upstream (Figure S2). In contrast, the 3′ portions of genes had relatively low frequencies of *Mu* insertions. Similar results were obtained using the MGSP's “filtered gene set”. *Mu* insertions occur in exons at much higher rates than in introns, which is consistent with a previous report using an engineered *Mu* transposon [7]. But not all exons have higher rates of insertion than introns. Indeed, the previously reported preference of *Mu* insertion of exons [7] can probably be explained simply by the preference of *Mu* to insert into 5′-most exons.

**Figure 2:**
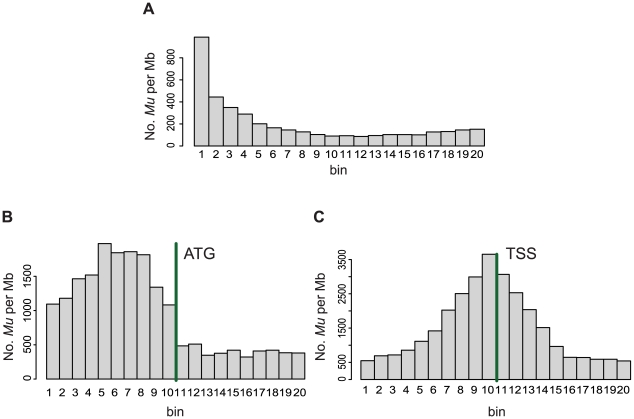
The distribution of *Mu* insertion sites within genes. (A) Sequences of genes (from annotated transcriptional start to poly-adenylation sites) were extracted from 15,050 full-length genes. Each gene sequence was divided into 20 equally sized bins. Because gene lengths differ, bin sizes differ from gene to gene. The x-axis lists these 20 bins 5′ to 3′. For each gene, the number of *Mu* insertions in each of the 20 bins was determined. Subsequently, the numbers of *Mu* insertions in each of the 20 bins and the lengths of each of the 20 bins were summed across the 15,050 genes. It was then possible to calculate the number of *Mu* insertions per Mb (y-axis) for each of the 20 bins. (B) 200-bp sequences around translation start sites (ATG, 200 bp left side and 200 bp right side) from each full-length gene were extracted and were divided into 20 bins, each of which was 20 bp in size. The x-axis lists these 20 bins 5′ to 3′. For each gene, the number of *Mu* insertions in each of the 20 bins was calculated. Subsequently, the numbers of *Mu* insertions in each of the 20 bins were summed across the 15,050 genes. The total summed length of each bin is 150,500 bp (20 bp bin length×15,050 genes). Using these data it was then possible to calculate the number of *Mu* insertions per Mb (y-axis) for each of the 20 bins. (C) 200-bp sequences around transcription start sites (TSS, 200 bp left side and 200 bp right side) from each full-length gene were extracted and were divided into 20 bins, each of which was 20 bp in size. The x-axis lists these 20 bins 5′ to 3′. For each gene, the number of *Mu* insertions in each of the 20 bins was calculated. Subsequently, the numbers of *Mu* insertions in each of the 20 bins were summed across the 15,050 genes. The total summed length of each bin is 150,500 bp (20 bp bin length×15,050 genes). Using these data it was then possible to calculate the number of *Mu* insertions per Mb (y-axis) for each of the 20 bins.

Further analyses were performed to understand the pattern of *Mu* insertion within genes without considering gene structure. Each gene, beginning at the transcription start site and ending at the polyA site, was split into 20 equally sized bins. The number of *Mu* insertions was counted in each of the 20 bins across all 15,050 genes. Figure 2A reveals a pronounced preference for insertion in the 5′-most bin and decreasing frequencies from 5′ to 3′. This pattern is observed even when using other numbers (viz., 10 and 50) of bins, indicating that *Mu* transposons exhibit a preference for insertion into the 5′ ends of genes.

To more specifically map the positions of preferred sites for *Mu* insertion, 400-bp sequences (200 bp each side) surrounding the transcription start sites (TSS) and translation start sites (ATG) were extracted from each of the 15,050 genes. The extracted 400-bp sequences were each divided into 20 bins and the numbers of *Mu* insertions in each bin counted and plotted, revealing that *Mu* exhibits a preference for regions 5′ of ATGs (Figure 2B) at or slightly 5′ of the TSS (Figure 2C).

### Non-uniform distribution of *Mu* insertions along chromosomes

**Figure 3:**
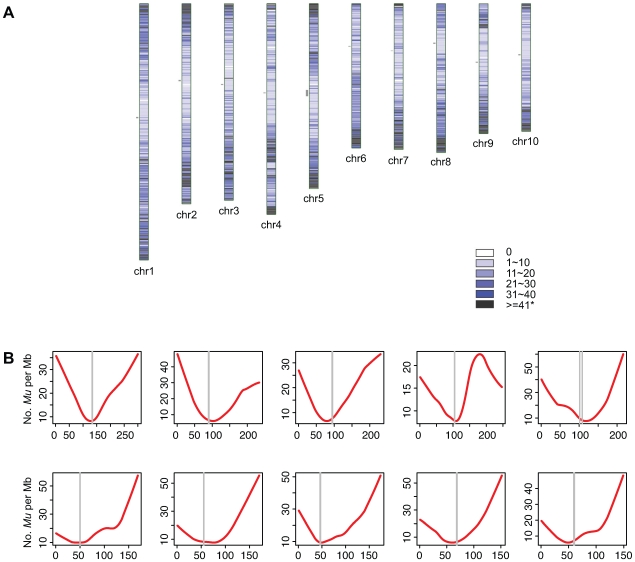
The distribution of *Mu* insertion sites in the maize genome. (A) Each horizontal line on chromosomes represents a 1-Mb window. Lines are intensity- and color-coded to indicate the number of *Mu* insertions per Mb. Grey vertical lines represent the approximate positions of centromeres [67]. (B) The locally-weighted polynomial regression (*LOWESS*) curve with smooth span (f) equaling to 0.4 of the number of *Mu* insertions per 1-Mb window (y-axis) was plotted versus the corresponding window's coordinates (Mb, x-axis). The vertical paired grey lines represent approximate centromere positions [67]. Those patterns we observed are unlikely to be artifacts of the removal of repetitive MFS, because only a small proportion of all MFSs (1.4%) were removed based on their ability to map to multiple positions in the genome.

Access to the B73 reference genome allowed us to examine the distributions of *Mu* insertions across chromosomes. Plotting numbers of *Mu* insertions per Mb reveals a non-uniform distribution on each of the chromosomes (Figure 3A). Chi-square tests provided strong evidence for non-uniformity on each chromosome (Pearson's 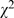 test, all p-values <2.2×10^−16^). To better visualize these non-uniform patterns of *Mu* insertion, *LOWESS* curves were plotted (Figure 3B) [22]. For each chromosome, a pronounced “bowl-like” trend was observed in which frequencies of *Mu* insertions are higher at the ends of chromosomes than in peri-centromeric regions.

Because this trend is reminiscent of the distribution of meiotic recombination events in maize and other species [23],[24],[25], we were interested in examining the genome-wide distribution of meiotic recombination events per Mb and comparing these distributions to those of *Mu* insertion. We generated a combined genetic map containing 10,143 sequence-based genic markers (Methods). The sequences of 6,362 of these genetic markers could be uniquely aligned to the B73 RefGen_v1 and have consistent positions on both the genetic and physical maps (Methods; Figure S3). Using data from Figure S3, rates of recombination per Mb were calculated for each 1-Mb window and *LOWESS* curves of rates of recombination per Mb were plotted versus the physical coordinates of the B73 RefGen_v1 (Figure S4).

Each chromosome exhibits a “bowl-like” pattern of recombination per Mb similar to the frequencies of *Mu* insertions per Mb, which is consistent with previous cytogenetic observations [26]. The similarity between the distributions of *Mu* insertions and recombination events is also evident at greater granularity; viz., the numbers of *Mu* insertions and meiotic recombination sites in 1-Mb bins are well correlated genome-wide (r = 0.6).

**Figure 4:**
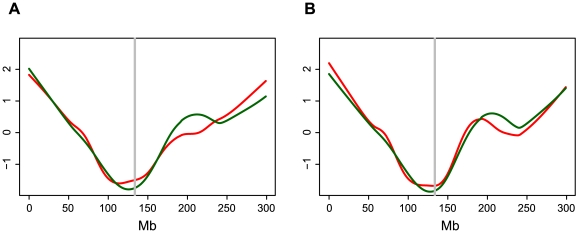
Number of *Mu* insertions and recombination rate (cM) per Mb corrected by gene number and gene length on chromosome 1. (A) Numbers of *Mu* insertions per gene per Mb (red line) and cM per gene per Mb (green line) are standardized as described in Methods. Locally-weighted polynomial regression (*LOWESS*) curves with smooth span (f) equaling to 0.4 for both standardized values were plotted against the physical coordinates of chromosome 1 (Mb, x-axis). The approximate centromere position is shown in grey [67]. (B) Numbers of *Mu* insertions per bp of genic sequence per Mb (red line) and cM per bp of genic sequence per Mb (green line) are standardized as described in Methods. Locally-weighted polynomial regression (*LOWESS*) curves with smooth span (f) equaling to 0.4 for both standardized values were plotted against the physical coordinates of chromosome 1 (Mb, x-axis). The approximate centromere position is shown in grey [67].

Because both meiotic crossovers and *Mu* insertions exhibit preferences for genes, we wondered whether the bowl-like patterns simply reflected gene density. To test this hypothesis we used the MGSP's “filtered gene set” to plot the number of genes (and bp of genic sequence) per Mb across the ten chromosomes. Similar “bowl-like” patterns were observed for the distributions of annotated genes/Mb and the proportion of genic DNA/Mb (data not shown). Even so, after expressing numbers of *Mu* insertions and recombination rates on a per gene or per bp of genic sequence basis, the bowl-like patterns persist (Figure 4, Figure S5, and Figure S6), demonstrating that the number of *Mu* insertions per gene (or per bp of genic regions) is generally greater at the ends of chromosomes than near the centromeres. Therefore, gene density can not *per se* fully explain the “bowl-like” patterns of *Mu* insertions and recombination.

### GC content of target sequences and a consensus sequence for *Mu* insertion sites

**Figure 5:**
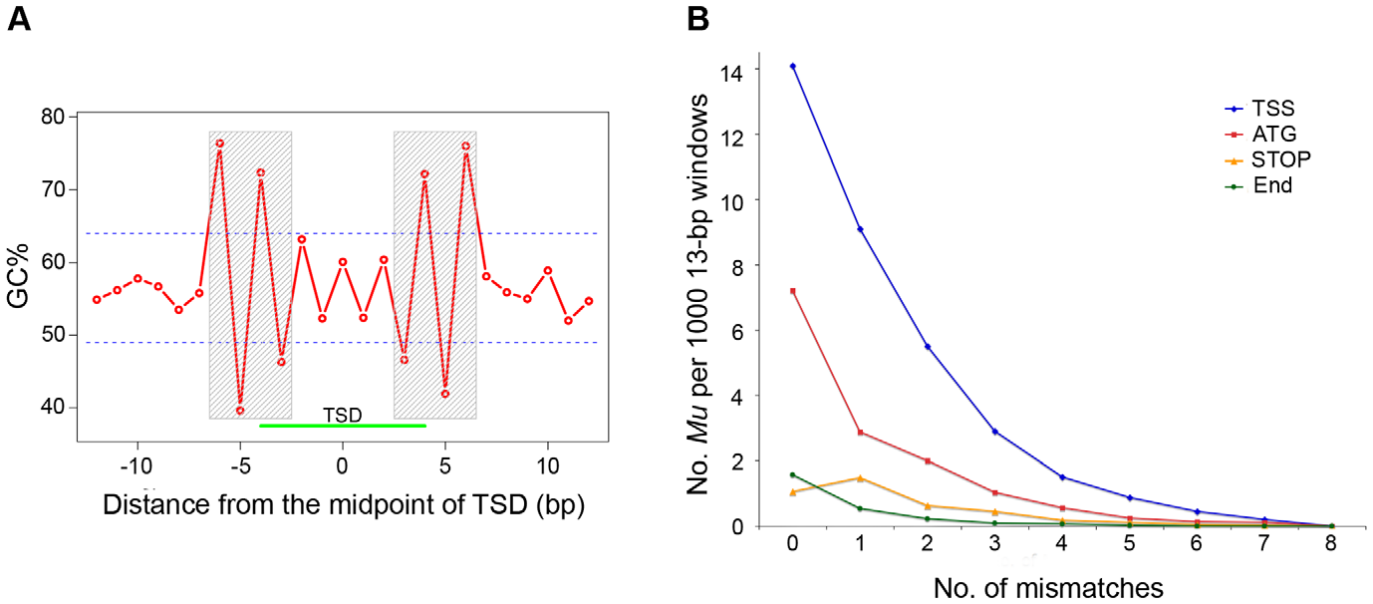
GC patterns at *Mu* insertion sites. (A) More than 2,000 non-redundant *Mu* insertion sites for which MFSs were available on both sides and exhibiting 9-bp TSDs were collected. The mid-point of each TSD was set as position 0. Relative positions decrease to the left and increase to the right. Average GC% was calculated for each position separately across >2,000 sequences and plotted against the relative positions. Regions between the dashed lines represent the 99% confidence interval of randomly sampling 10,000 GC percentages (Methods). The shaded boxes cover positions with GC% that differ significantly from expected by chance (Methods). Boxed regions are hypothesized to be *Mu* transposase binding sites. (B) 101-bp sequences centered at transcription start sites (TSS), translation start sites (ATG), translational end sites (STOP) and transcriptional end sites (END) were extracted from over 15,000 full-length genes, respectively. All 13-bp windows sliding from 1 to the end of each 101-bp sequence was compared to the consensus sequence: “SW::SWNNNNNWS::WS”. The number of mismatches was computed and sequences of these windows were assigned to nine groups containing 0–8 mismatches (x-axis). 13-bp sequences of *Mu* insertion sites were categorized into these nine groups as well. The frequency of sequences with *Mu* insertions (y-axis) in each group was plotted for four sets of sequences (TSS, ATG, STOP and END) respectively.

Weak consensus sequences for *Mu* insertion sites have been identified by several groups [7],[10],[27],[28]. To extend these results, we identified 2,217 non-redundant *Mu* insertion sites for which both the left and right MFSs were available and at which the expected 9 bp TSD could be detected. The mid-point of the TSD was set as position zero. The TSD is located at positions −4 to +4. GC content across the 2,217 sequences was calculated for each position (−12 through +12) separately. The null hypothesis that the GC content at each position does not differ from random can be rejected for positions −6 to −3 and +3 to +6 (p-values<0.01; Methods) (Figure 5A). The consensus sequence for position −6 to +6 is “SW::SWNNNNNWS::WS” (consistent with terminology of Dietrich *et al.* 2002 [10], the TSD is flanked by pairs of double colons), where S represents G or C, W represents A or T, and N can be any base. During *Mu* insertion the 9-bp TSD is thought to arise via the introduction and subsequent repair of staggered single-strand cuts before and after positions −4 and +4, respectively. These cuts are presumably generated by the *Mu* transposase. Position −6 to −3 and +3 to +6 have the pattern “SW::SW” and “WS::WS” (subsequently referred to as SWSW), respectively. We therefore hypothesize that SWSW is the preferred sequence of the *Mu* transposase binding/cutting site.

Although SWSW is the preferred pattern, it is not required for *Mu* insertion, because only 61 of the 2,127 *Mu* insertion sites have the exact “SWSW” pattern. The 2,127 *Mu* insertions are a subset of the whole set of *Mu* insertions (N = 42,948). The remaining *Mu* insertion data (N = 40,821) were used to cross-validate the consensus. In this independent data set, 13-bp windows surrounding *Mu* insertion sites with fewer mismatches relative to the consensus were over-represented (data not shown).

To rule out the possibility that an ascertainment bias explains these results, i.e., that “SWSW” is simply enriched in those genic regions that experience the highest frequencies of *Mu* insertions (e.g., promoters or 5′-UTRs), similar analyses were performed on regions surrounding certain genic landmarks: viz., transcription start sites (TSS), translation start sites (ATG), translation stops sites (STOP) and transcriptional end sites (END) from the flcDNA genes (Figure 5B, Methods). Surrounding each of these genic landmarks, the frequency of *Mu* insertions generally decreases as the number of mismatches increases. Interestingly, even after controlling for the number of mismatches in the 13-bp window, frequencies of *Mu* insertions are highest in the TSS, demonstrating that the TSS is enriched for *Mu* insertions for reasons other than having a high frequency of the putative transposase binding sites. Hence, the distribution of the consensus sequence is not sufficient to explain the non-uniform distribution of *Mu* insertion sites within genes.

### Epigenetic modifications are correlated with frequencies of *Mu* insertions

It has been hypothesized that frequencies of *Mu* insertions are associated with chromatin structure [7],[29]. To test this hypothesis, frequencies of *Mu* insertions in single-copy regions of the entire genome associated with various types of histone modifications (Table 2, Methods) were compared. The average number of *Mu* insertions per Mb was significantly greater for regions that contained H3K4me3 modifications than for regions that contained no H3K4me3 modification (Wilcoxon rank-sum *p*-value <0.0001). The same held true for H3K9ac and H3K36me3 modifications. However, for H3K27me3 modification, the situation was reversed in that the presence of H3K27me3 modifications was associated with a statistically significant decrease (Wilcoxon rank-sum *p*-value <0.0001) in the average number of *Mu* insertions per Mb.

**Table 2:** *Mu* insertions in single-copy regions with different epigenetic modifications.

	H3K4me3^1^	H3K9ac^2^	H3K36me3^3^	H3K27me3^4^	Meth^5^	MF^6^	WGS-GSS^7^
Total length (Mb)	34.3	44.2	31.2	7.7	15.5	77.7	11.3
No. *Mu*	22,871	24,866	15,552	599	156	21,864	623
No. *Mu*/Mb	668	563	499	78	10	281	55

1 CHIP-seq of trimethylation of lysine 4 in histone 3 [13].

2 CHIP-seq of acetylation of lysine 9 in histone 3 [13].

3 CHIP-seq of trimethylation of lysine 36 in histone 3 [13].

4 CHIP-seq of trimethylation of lysine 27 in histone 3 [13].

5 DNA methylation (McrBC sensitive, [13]).

6 Methylation filtration (hypomethylated, [12]).

7 Whole genome shotgun (WGS) - genome survey sequences (GSS) as per [66].

To check for possible interactions among histone modifications with respect to *Mu* insertions, a linear model with the number of *Mu* insertions per Mb as the response variable and the presence or absence of each histone modification and all possible interactions as explanatory variables was fit to the data. Each term in the model was significant at the 0.01 level except for two of the four three-way interactions and the four-way interaction among the four indicator variables corresponding to the four histone modifications. Thus, there is good evidence that the effects of the histone modifications on *Mu* insertion rates are not simply additive.

Table S2 shows that average number of *Mu* insertions per Mb for all 16 possible combinations of the four histone modifications. A second linear model was fit to the data used to generate Table S2. This model allowed each of the 16 possible histone modification patterns to have a different underlying *Mu* insertion rate. After testing for differences between each pair of patterns using the Tukey-Kramer method [30] for all pairwise comparisons, many significant differences across histone modification patterns were identified. In particular, regions with all histone modifications except H3K27me3 had a significantly higher average number of *Mu* per Mb than each of the other 15 patterns. Generally speaking, the H3K9ac or H3K4me3 modifications were most associated with elevated frequencies of *Mu* insertions among four examined histone modifications. H3K27me3 regions had relatively low frequency of *Mu* insertions even when other modifications were co-located. In contrast, H3K36me3 regions with H3K4me3 and/or H3K9ac co-located had much higher frequencies of *Mu* insertions than did H3K36me3 regions without H3K4me3 and/or H3K9ac co-located (Table S2). This illustrates one example of the type of interaction identified in our initial linear model analysis.

We also examined the relationship between DNA methylation and *Mu* insertion rates. This was done by identifying single-copy regions that either have evidence of containing DNA methylation (McrBC sensitive) or evidence of being hypomethylated (methylation filtration data). Regions with evidence of being methylated and being hypomethylated have frequencies of *Mu* insertions per Mb 5× lower or 5× higher than the WGS-GSS control, indicating that at least in single-copy regions methylation is strongly negatively correlated with frequencies of *Mu* insertions.

## Discussion

The stocks used in this study exhibited a high rate of *Mu* element transposition based on standard Robertson's seedling test assays [31]. Consequently, most *Mu* insertions investigated in this study were recently generated. Most of them are far less likely to have been subjected to selection than are less active elements. Hence, their distributions are expected to better reflect insertion site preferences than do the distributions of “ancient” transposons detected during genome sequencing projects.

Although there are some exceptions (viz., the long arms of Chr 2 and 5), overall the distributions of *Mu* insertions and meiotic recombination are similar, suggesting that common features may be involved in site selection for both kinds of events. Both types of events cluster in genes (this study and [32]), but we have demonstrated that gene density is not sufficient to explain the non-uniform distributions of *Mu* insertions and recombination events along chromosomes.

Both types of events share a preference for GC-rich regions. Meiotic recombination events cluster in GC-rich regions in humans and yeast [33],[34]. Similarly, our data are consistent with a prior report [7] that *Mu* insertion sites exhibit a bias toward GC-rich regions. The average GC content of the 100-bp intervals surrounding *Mu* insertion sites is 56% in our data set versus the average 47% GC in the filtered gene set. In addition, like the P elements of *Drosophila* [35],[36] to which they have been compared mechanistically [37], *Mu* transposons exhibit a strong preference for 5′-ends of genes, which are typically enriched for GC relative to 3′ ends [38].

What is not clear from these data is whether the high GC content of preferred *Mu* insertion sites is a cause or an effect of *Mu* insertion site preference. The finding that the preferred *Mu* insertion site consensus sequence does not exhibit a strong GC signal (50% GC) suggests that although *Mu* transposon exhibits a preference for regions that happen to have a high GC content, they do so for reasons other than the GC content of these regions. The overall frequency of *Mu* insertions in a given sequence is related to its similarity to the preferential consensus sequence (SW::SWNNNNNWS::WS). However, in different genic contexts (e.g., 5′-UTRs, 3′-UTRs) this preferential sequence has dramatically different impacts on *Mu* insertion frequency, strongly indicating that *Mu* insertion site selection is more dependent on genic context than on DNA sequences *per se*.

If DNA sequences are not a major factor in site selection for *Mu* insertions, what are the causal factor(s)? Frequencies of *Mu* insertions and meiotic recombination are both low in pericentromeric regions, which are rich in heterochromatin, suggesting an association with chromatin structure. Indeed, our genome-wide results demonstrate that various types of epigenetic modifications are differentially correlated with frequencies of *Mu* insertions. In addition, the 5′-ends of genes, which are preferred sites for both *Mu* insertion in maize and meiotic recombination in yeast and maize [25],[33],[34],[35],[36] have distinctive epigenetic modifications. We demonstrated that *Mu* insertions particularly favor regions surrounding the TSS, which also exhibit strong signal for both H3K4me3 and H3K9ac [13],[39] and exhibit low levels of cytosine methylation [13],[40],[41]. In addition, H3K4me3 modifications which cluster at DSB hotspots in yeast [42] were highly correlated with *Mu* insertions in this study. Both of these epigenetic modifications are associated with open chromatin structure [39],[40],[43],[44],[45],[46]. Hence, it is likely that chromatin structure plays a key, and perhaps even a causal role, in site selection for both *Mu* insertion and meiotic recombination. A number of other transposons (e.g., *Ac*/*Ds*, P elements, MITEs, and *Tos17*) exhibit preferences for low-copy genic regions [3],[29],[35],[47],[48],[49],[50],[51], which cluster in the euchromatin. It is therefore possible that these transposons may share a common mechanism of insertion site selection. To further test the hypothesis that chromatin structure is a common feature driving transposon insertion site preferences, the co-localization of new transposon insertion sites and/or transposase binding site with epigenetic marks could be assayed in mutants such as *mop1* [52] that would be expected to alter the distribution of epigenetic marks.

## Methods

### Genetic stocks

The *Mu* activity of *Mu* stocks was determined using Robertson's standard seedling test assays [31] that detects the effects of *Mu* transposition via the appearance of new mutations. The progeny of crosses between *Mu* active lines and various inbreds and hybrids were self-pollinated to produce the *Mu* stocks used in this study. The resulting kernels were planted and leaves harvested for genomic DNA isolation. *Mu* flanking sequences were amplified from these genomic DNAs via DLA [11].

Genetic mapping was conducted using (N≤357) of the IBM (Intermated B73×Mo17) Recombinant Inbred Lines (RILs) as shown in Figure S7 and listed in Table S3. The IBM RILs are the standard mapping population for the maize genetics community.

### DLA for amplification, primer design, and barcode design

DLA-454 was conducting following the protocol in [11]. As compared to Liu *et al.*, additional barcode primers were used:

AaMu

5′ GCCTCCCTCGCGCCATCAGTCTGAGGCCTCYATTTCGTCGAATC

AbMu

5′ GCCTCCCTCGCGCCATCAGGTTAGCGCCTCYATTTCGTCGAATC

AcMu

5′ GCCTCCCTCGCGCCATCAGGGTACTGCCTCYATTTCGTCGAATC

AdMu

5′ GCCTCCCTCGCGCCATCAGCATGTGGCCTCYATTTCGTCGAATC

AhMu

5′ GCCTCCCTCGCGCCATCAGATTCTGGCCTCYATTTCGTCGAATC

### Data processing of *Mu* 454 reads

#### Categorization of reads based on barcodes and trimming

Raw 454 reads were categorized by their barcodes. SeqClean (http://compbio.dfci.harvard.edu/tgi/software/) was used to trim barcodes, primers and partial *Mu* TIR sequences. A two-step trimming strategy was applied. First, the *Mu* primer and adaptor primer (default overlapping requirement, ≥80% identity with primers) were removed. Trimmed sequences with the size ≥60bp were subjected to a second round trimming to remove *Mu* TIR regions (≥30 bp overlapping, ≥80% identity with known, novel and putative pTIRs (34 bp)).

#### Mapping MFS to the B73 reference genome (B73 RefGen_v1)

Trimmed MFSs with size ≥40 bp were aligned to the B73 RefGen_v1 using BLASTN. Only if there was a single best BLAST alignment (lowest E-value hit) with ≥95% identity and ≥40 bp covering the 5′ region of the query MFS, was a MFS considered to be unambiguously mapped onto the genome, which allowed the corresponding *Mu* insertion site to be determined. The requirement for a 5′ match was established because the exact *Mu* insertion sites were inferred by aligning the first 5′ bases of MFSs to the reference B73 genome. Failure of MFSs to align to the B73 RefGen_v1 could be due to DNA sequence polymorphisms between B73 and the *Mu* stocks and/or DNA sequencing errors and/or incomplete trimming.

#### Identification of SNPs between MFSs and the B73 RefGen_v1

SNPs were identified within unique alignments of MFSs and the B73 RefGen_v1. For individual B73 genomic regions where multiple MFSs were mapped, MOSAIK, a reference-guided read aligner, was used to conduct realignment of all sequences in the region (≥95% identity) (http://bioinformatics.bc.edu/marthlab/Mosaik) and SNPs were identified using an updated version of PolyBayes [53] (http://bioinformatics.bc.edu/marthlab/PolyBayes). SNP calls required at least two 454 reads of support and the proportion of reads with a given SNP among the total reads that cover a SNP site must have been ≥20%. The probability of called SNPs being correct (as calculated by PolyBayes) was larger than 0.9. Among the ∼1.5 Mb of the B73 RefGen_v1 with at least 2× MFSs coverage, 19,252 SNPs were identified. These SNPs were used to distinguish among unique *Mu* insertions at the same genomic positions.

#### Determination of non-redundant *Mu* insertions

To conservatively correct for errors in trimming MFS 454 reads, insertion sites with the same orientation that clustered within 3 bp of each other were regarded as the same site. Those MFS with different orientations could be the result of the same or different insertion events. Therefore, if the distance between two sites was 8-bp, indicating a 9-bp target site duplication (TSD), they were counted as paired insertions. Otherwise, they were regarded as different insertions.

MFSs were further distinguished using TIR and SNP data if multiple reads were aligned to the same insertion sites. Different types of TIRs indicate independent insertions from different genetic lines. To avoid inappropriately separating MFSs at the same position based on sequence errors in TIRs, additional criteria were used. MFSs were treated as independent insertion events if either of the criteria below was satisfied:

- Both TIRs and SNPs provide evidence of independence.
- A TIR was supported by at least two reads and the total number of reads containing this TIR is not less than 20% of the total reads at this site.

#### Identification of novel *Mu* TIRs

An edit distance between two sequences is the minimum number of base modifications, including base substitution, deletion and insertion, required to change one sequence into the other sequence [14]. The first 34-bp sequence was extracted from each of 454 reads after the first step of trimming. Edit distances were calculated between the 34-bp sequence and all known 34-bp pTIRs. The smallest edit distance was obtained for this 34-bp sequence versus all known pTIRs. This procedure was repeated for additional 34-bp windows obtained by starting at nucleotide positions 2 to 7. Among seven smallest edit distances from seven repeats of each read, the smallest value was considered the minimum edit distance (MED) between possible pTIRs and known pTIRs. If the MED was between 2 and 10, the corresponding 34-bp sequence was extracted and treated as the putative pTIR. In total, 153,136 reads with unknown pTIRs were obtained. After removing redundancy, 292 non-redundant putative pTIRs that had at least 100 reads of support were obtained and imported to Sequencher 4.7 for sequence clustering. Finally, 21 novel pTIRs were obtained.

#### Calculation of a cut-off to identify *Mu* insertion hotspot genes

A total of 31,838 unique *Mu* insertions were mapped onto 32,477 annotated genes (including 500 bp extensions upstream and downstream from the transcription start and poly-adenylation sites). We randomly assigned each of the 31,838 *Mu* insertions to one of the 32,477 genes. When making each random assignment, each gene was equally likely to receive an insertion. This simulates a scenario in which no genes are preferential targets for insertion. After all assignments were made, we noted the maximum number of insertions received by any one gene. The entire process was repeated 10,000 times to obtain 10,000 maximum numbers of insertions. For 95% of the 10,000 simulation runs, the maximum number of insertions in any one gene was less than 9. Thus, we declared genes with 9 or more insertions to be hotspot genes.

Approximately 1% [4],[54] or fewer [37] of gametes carry deletions adjacent to a given *Mu* insertion. Such events could potentially cause mis-classifications of the same insertion as two (or more) independent events. To determine if this rare event could account for the observed hotspots. All of the *Mu* insertions at eight hotspot genes were manually checked for independence using data from the pTIRs, SNPs in the MFSs and TSDs. These analyses found no evidence that *Mu* insertion hotspots artifacts caused by *Mu* adjacent deletions.

#### mRNA-seq of B73 SAMs

Shoot apical meristems (SAMs) L1 and L2 were harvested from 14-day B73 seedlings. SAMs were fixed, embedded in paraffin, sectioned and tissues collected via LCM as described previously [55]. L1 and L2 were collected separately from the same SAMs. RNA extraction, amplification and synthesis of double-stranded cDNA were conducted according to previous procedures [55].

#### mRNA-seq of B73×Mo17 hybrid seedlings

RNAs of B73×Mo17 hybrids (F_1_) were extracted from 14-day-old F_1_ seedlings. RNAs were purified using DNaseI treatment followed by cleanup with the RNeasy Plant Mini Kit (Qiagen, Valencia, CA) as per manufacturer instructions. Sequencing library construction was constructed using the Illumina mRNA-Seq sample preparation kit.

#### Alignments of mRNA–seq reads

Illumina reads were aligned to the B73 RefGen_v1 [18] with the short read aligner NOVOALIGN (http://www.novocraft.com). One and two mismatches per read were allowed in B73 and F_1_ RNA-seq data, respectively. Only reads that uniquely mapped to the B73 RefGen_v1 were considered for further analysis.

#### Generation of the full-length gene set

A total of 22,490 full-length cDNAs [21] were mapped to the B73 RefGen_v1 using GMAP [56]. Alignments were accepted only if a cDNA mapped to a single location with ≥95% identity for all exons, ≥90% cDNA coverage and ≤60 bp tail lengths. Gene structure was defined by the original annotation in combination with the GMAP splice-alignment results.

#### Statistical test of significant GC content at *Mu* insertion sites

2,127 highly reliable non-redundant *Mu* insertion sites with both sides of MFSs and 9 bp TSDs were used to study the sequence characteristics of *Mu* insertion sites (e.g. GC content). All sequences were aligned at the mid-point of the TSD, which was set as position 0. Relative positions decreased to the left and increased to the right. Average GC content was calculated at each specific position separately across 2,127 sequences. To obtain the probability of observed GC% at each given position, one base was randomly extracted from each of the 2,127 *Mu* insertion sites. An insertion site for purposes of this analysis is a 400 bp region centered on the 0 position of the TSD. Mean GC% was computed across all extracted nucleotides (2,127 bases). This procedure was repeated 10,000 times to obtain 10,000 GC% values. 99% of these 10,000 GC% values were between the dashed lines on Figure 4A. GC percentages falling outside this range were considered significant.

#### Consensus analysis of *Mu* insertion sites in different genic regions

101-bp sequences centered at transcription start sites (TSS), translation start sites (ATG), translation stops sites (STOP) and transcriptional end sites (END) were extracted from each of the 15,050 flcDNA genes. The number of mismatches of 13-bp-window sequence compared with “SW::SWNNNNNWS::WS” was computed and sequences of all sliding windows (1-bp stepping) from 101-bp sequences were categorized to nine groups with 0–8 mismatches, respectively. 13-bp of *Mu* insertion sites presumably centered at the midpoints of TSDs were categorized to these nine groups as well. The frequency of *Mu* insertions of each group was plotted (Figure 5B).

### Recombination and *Mu* insertion

#### Construction of an integrated genetic map

An integrated genetic map of maize was constructed using 10,188 markers generated by multiple mapping projects (Missouri Mapping Project [57],[58], Genoplante [59], and our ISU mapping project ([60],[61] and unpublished work). All of these markers had been used to genotype overlapping sets of recombinant inbred lines (RILs) from the IBM population (Figure S7). As part of this study some of the ISU IDP markers were used to genotype additional IBM RILs (Figure S8). All downloaded and newly generated genotyping scores were analyzed using the MultiPoint mapping software (Initial threshold recombination rate: 0.05; Final threshold recombination rate: 0.35; Kosambi function for genetic distances calculation) [62],[63]. The genetic positions of 10,143 (∼99%) mapped markers (Table S4) were quality checked with previous map builds to repair inconsistencies.

#### Mapping genetic markers to physical positions

Most of the genetic markers included in the integrated genetic map were developed from known sequences. The source sequences (genomic DNAs and ESTs) of these mapped markers were trimmed using Seqclean against the UniCore vector database (dated: April 22nd, 2008). Sequences of 9,127 markers were successfully mapped to the B73 RefGen_v1 genome reference sequence via BLASTN (≥99% identity, ≤20 bp tails) and GMAP (≤10 kb intron span, ≥95% identity of all exons, ≥90% coverage, ≤5% tails or ≤60 bp tails) for the genomic DNAs and ESTs, respectively. Only sequences that mapped to a single location in the genome were used for subsequent analyses (N = 7,185). For the purposes of this analysis, we assumed that the structure of the B73 reference genome was correct. Hence, 823 markers that aligned to the B73 RefGen_v1 were removed because they had genetic positions that were inconsistent with the reference genome sequence. It is, however, expected that in subsequent analyses these currently excluded genetic markers may be useful in improving the structure of the B73 reference genome. Using the pipeline described above, 6,362 of the markers could be anchored to the B73 RefGen_v1.

#### Estimation of genetic positions

The 6,362 genetic markers that were anchored to the reference genome were used to draw physical-genetic curves for each chromosome by fitting a General Additive Model (GAM) using the gam function of R [64],[65]. The fitted curve was then used to estimate genetic positions based on physical positions for estimating recombination rates.

#### Estimation of recombination rates (cM/Mb)

Each chromosome was divided into non-overlapping 1-Mb bins. The genetic positions of the starting and ending positions (expressed in cM) of bins were estimated based on the fitted physical-genetic curves. The physical positions of the starting and ending positions of bins (expressed in Mb) were determined based on the B73 reference genome. The estimated recombination rate of a bin (cM/Mb) is calculated by dividing the genetic length of the bin expressed in cM by the physical length of the bin expressed in Mb (physical length).

#### Correction of recombination rates and *Mu* insertion frequencies by gene numbers and genic lengths

Numbers of *Mu* insertions and genetic length (cM) were determined in each of non-overlapping 1-Mb bins in each chromosome of the reference genome. The number of genes and the total genic length (bp) were calculated for the each 1-Mb bin as well. Numbers of *Mu* insertions per gene (or bp) per Mb and cM per gene (or bp) per Mb were then computed as the gene numbers (or genic lengths) corrected *Mu* insertion frequencies and recombination rates, respectively.

#### Standardization of recombination rates and *Mu* insertion frequencies

To visualize recombination rates and *Mu* insertion frequencies on the same plot, both values were standardized. Values were standardized using the formula: 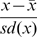, where 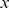 is either recombination rate (cM/Mb) or *Mu* insertion frequency (number of *Mu* insertions per Mb). 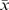 represents the mean of all 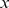 values in the same chromosome; 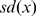 represents the standard deviation of these 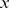 values. Gene and genic length corrected recombination rates and *Mu* insertion frequencies were standardized using the same method.

### Epigenetic modifications and *Mu* insertion

The positions of histone modifications (H3K4me3, H3K9ac, H3K36me3 and H3K27me3) and cytosine methylation relative to BAC sequences from [13] were downloaded from http://www.ncbi.nlm.nih.gov/geo/ and mapped to the B73 RefGen_v1 (100% identity, 100% coverage). The 80–90% of sequences from each data set that uniquely mapped to the genome were used for further analysis. Whole genome shotgun genomic survey sequences (WGS-GSS, N = 17,232) and methylation filtration (MF) sequences (N = 349,950) [12] (http://magi.plantgenomics.iastate.edu/) [66] were mapped to the B73 RefGen_v1 (≥98% identity, ≥95% coverage). Only reads with a single best alignment (lowest e-value) were considered for the further analysis, resulting in the mapping of 81% (13,938/17,232) and 87% (304,490/349,950) of the WGS-GSS and MF reads, respectively.

### Accession numbers

Sequence read archive accession no: SRX007377, SRX007378.

## Supporting Information

Figure S1 Clustering of 454 MFSs via alignment to the B73 RefGen_v1. Reads were categorized by their barcodes. A two-step trimming strategy was applied to remove barcodes, primers and amplified TIR sequences. Trimmed MFSs were mapped to the B73 RefGen_v1. Alignments were required to exhibit ≥95% identity, ≥40 bp overlap and have no 5′ tails that failed to align to the B73 RefGen_v1. Only reads with a single best hit (lowest e-value) were used for further analyses (Methods). Finally, redundancy was removed to obtain a non-redundant set of *Mu* insertions.
(1.50 MB TIF)

Figure S2 Distribution of *Mu* insertion sites in different gene regions. The numbers of *Mu* insertions were calculated for eight different genic regions (100 bp upstream of the TSS: purple; the 5′ UTR: orange; 5′-most exons, all internal exons [as a group], and 3′-most exons: blue; all introns [as a group]: black lines) of the ∼12,000 full-length genes (excluding single-exon genes from the 15,050 full-length gene set). These regions are listed on the x-axis. For each gene, the number of *Mu* insertions in each of the eight genic regions was determined. Subsequently, the numbers of *Mu* insertions in each of the eight genic regions and the lengths of each of eight genic regions were summed across the 15,050 genes. It was then possible to calculate the number of *Mu* insertions per Mb (y-axis) for each of the eight genic regions. A Pearson's Chi-square test was used to test the null hypothesis that the probability of an insertion in each genic region is proportional to its total length. This null hypothesis was rejected (p-value <2.2e-16), providing strong evidence that frequencies of *Mu* insertion vary across genic regions.
(0.40 MB EPS)

Figure S3 Genetic-physical map of 6,362 genetic markers. The genetic position (cM) of each marker was plotted against its physical coordinates on the 10 chromosomes of the B73 reference genome (Mb) (Methods). Approximate centromere positions (Wolfgruber *et al.*, [67]) are flanked by pairs of vertical grey lines. Those chromosomal regions with a paucity of polymorphic genetic markers are, based on comparative genomic hybridization (CGH) data (from Springer *et al.*, *PLoS Genetics *2009 [doi:10.1371/journal.pgen.1000734]), highly conserved between B73 and Mo17. The log intensity ratio of Mo17 to B73 (log2(Mo17/B73), y-axis) for each CGH probe is plotted versus its physical position on the B73 RefGen_v1 (Mb). CGH probes with statistically significant values of log2(Mo17/B73) (q-value<0.05) are indicated in red (Mo17>B73) and blue (B73>Mo17), while non-significant probes are indicated in green (B73 = Mo17).
(2.51 MB TIF)

Figure S4 The distribution of recombination events on reference chromosomes. The recombination rate was estimated for every non-overlapping 1-Mb window across the genome (Methods). The locally-weighted polynomial regression (LOWESS) curve with smooth span (f) equaling to 0.4 was plotted (green line) using the recombination rate (cM) per 1-Mb window (y-axis) versus the corresponding window's coordinates on the B73 reference chromosome (Mb, x-axis). Approximate centromere positions from Wolfgruber et al. [67] are shown in grey.
(0.33 MB EPS)

Figure S5 Numbers of *Mu* insertions and recombination rates per Mb corrected by numbers of genes on reference chromosomes 2–10. Numbers of *Mu* insertions per gene per Mb (red lines) and cM per gene per Mb (green lines), respectively were standardized as described in Methods. The locally-weighted polynomial regression (LOWESS) curves with smooth span (f) equaling to 0.4 of both standardized values were plotted against the physical coordinates (Mb, x-axis) on reference chromosomes 2–10. Approximate centromere positions from Wolfgruber et al. [67] are shown in grey.
(0.38 MB EPS)

Figure S6 Numbers of *Mu* insertions and recombination rates per Mb corrected by bp of genic sequences on reference chromosomes 2–10. Numbers of *Mu* insertions per bp of genic sequence per Mb (red lines) and cM per bp of genic sequence per Mb (green lines), respectively were standardized as described in Methods. The locally-weighted polynomial regression (LOWESS) curves with smooth span (f) equaling to 0.4 of both standardized values were plotted against the physical coordinates (Mb, x-axis) on reference chromosomes 2–10. Approximate centromere positions from Wolfgruber et al. [67] are shown in grey.
(0.38 MB EPS)

Figure S7 IBM RILs used to generate various genetic maps. Venn diagram shows numbers of IBM RILs used in each of several genetic mapping studies (Table S3). Data from these mapping projects, supplemented with additional data (Figure S8), were used to construct the integrated genetic map presented in Table S4.
(0.48 MB EPS)

Figure S8 Markers used to construct the integrated genetic map. An integrated genetic map of maize was constructed based on genotyping data from 10,143 markers from multiple mapping projects (Missouri Mapping Project (MMP) (Coe et al., Plant Phys 2002, Cone et al., Plant Phys 2002), Genoplante (Falque et al., Genetics 2005), ISU-IDP/TIDP (Map 7) (unpublished), ISU SNP (Liu et al., Genetics 2010)). Some IDP markers were used to genotype additional IBM RILs as part of this study. This flowchart provides types and numbers of markers used to genotype RILs. See also Figure S7 and Tables S3 and S4.
(0.56 MB TIF)

Table S1 Novel pTIRs.
(0.10 MB DOC)

Table S2 Frequencies of *Mu* insertions in different combinations of four epigenetic modifications in histone 3.
(0.05 MB DOC)

Table S3 List of 357 RILs in the integrated genetic map.
(0.38 MB DOC)

Table S4 10,143 genetic markers in the integrated map.
(9.95 MB XLS)
