# Supplementary figures and images for: Mu Transposon Insertion Sites and Meiotic Recombination Events Co-Localize with Epigenetic Marks for Open Chromatin across the Maize Genome
Source: PLoS Genet. 2009 Nov 20;5(11):e1000733. doi: 10.1371/journal.pgen.1000733 (PMC2774946; doi:10.1371/journal.pgen.1000733)

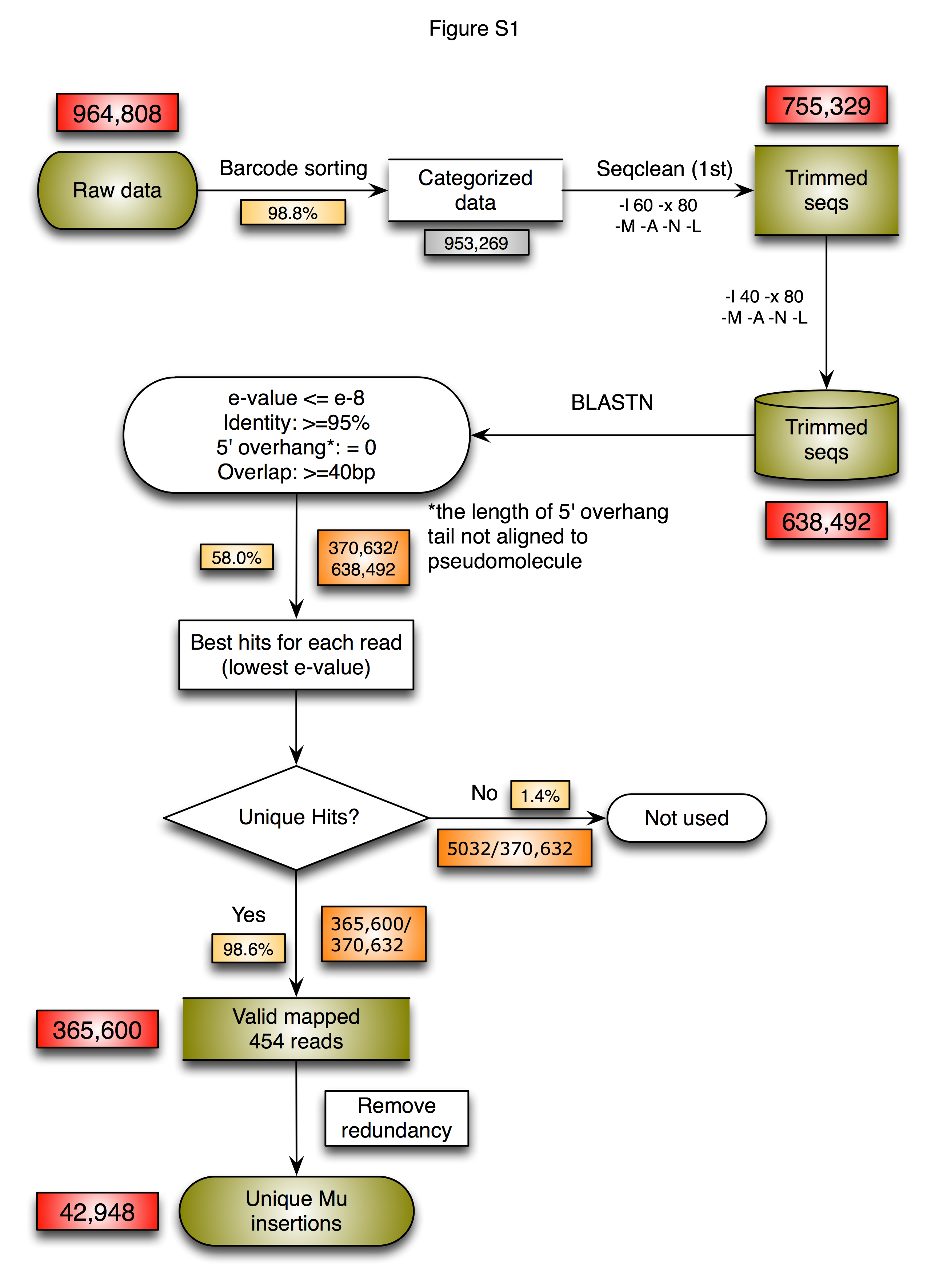

Supplement: Figure S1 — Clustering of 454 MFSs via alignment to the B73 RefGen_v1. Reads were categorized by their barcodes. A two-step trimming strategy was applied to remove barcodes, primers and amplified TIR sequences. Trimmed MFSs were mapped to the B73 RefGen_v1. Alignments were required to exhibit ≥95% identity, ≥40 bp overlap and have no 5′ tails that failed to align to the B73 RefGen_v1. Only reads with a single best hit (lowest e-value) were used for further analyses (Methods). Finally, redundancy was removed to obtain a non-redundant set of Mu insertions. (1.50 MB TIF) [file pgen.1000733.s001.tif]

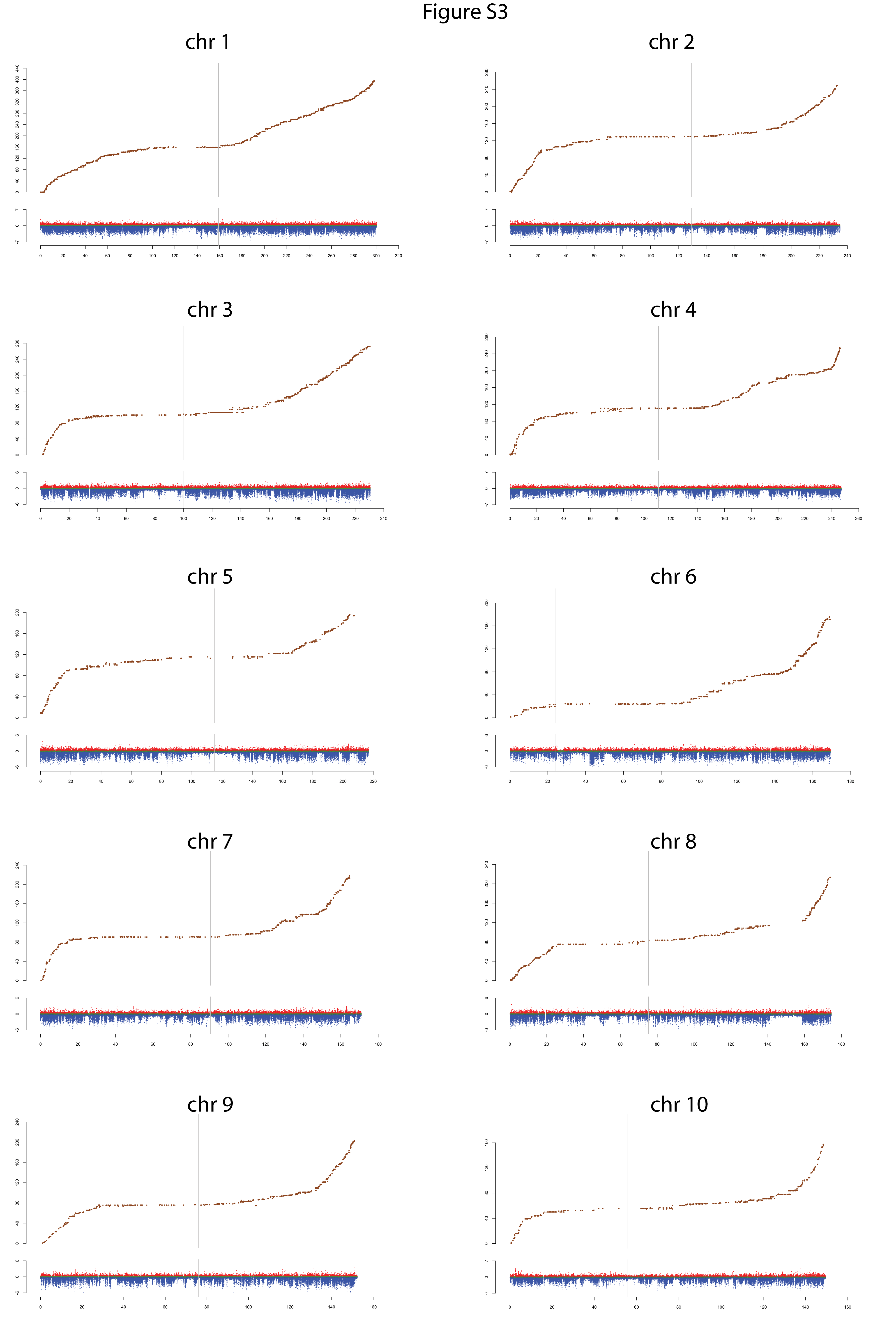

Supplement: Figure S3 — Genetic-physical map of 6,362 genetic markers. The genetic position (cM) of each marker was plotted against its physical coordinates on the 10 chromosomes of the B73 reference genome (Mb) (Methods). Approximate centromere positions (Wolfgruber et al., [67]) are flanked by pairs of vertical grey lines. Those chromosomal regions with a paucity of polymorphic genetic markers are, based on comparative genomic hybridization (CGH) data (from Springer et al., PLoS Genetics 2009 [doi:10.1371/journal.pgen.1000734]), highly conserved between B73 and Mo17. The log intensity ratio of Mo17 to B73 (log2(Mo17/B73), y-axis) for each CGH probe is plotted versus its physical position on the B73 RefGen_v1 (Mb). CGH probes with statistically significant values of log2(Mo17/B73) (q-value<0.05) are indicated in red (Mo17>B73) and blue (B73>Mo17), while non-significant probes are indicated in green (B73 = Mo17). (2.51 MB TIF) [file pgen.1000733.s003.tif]

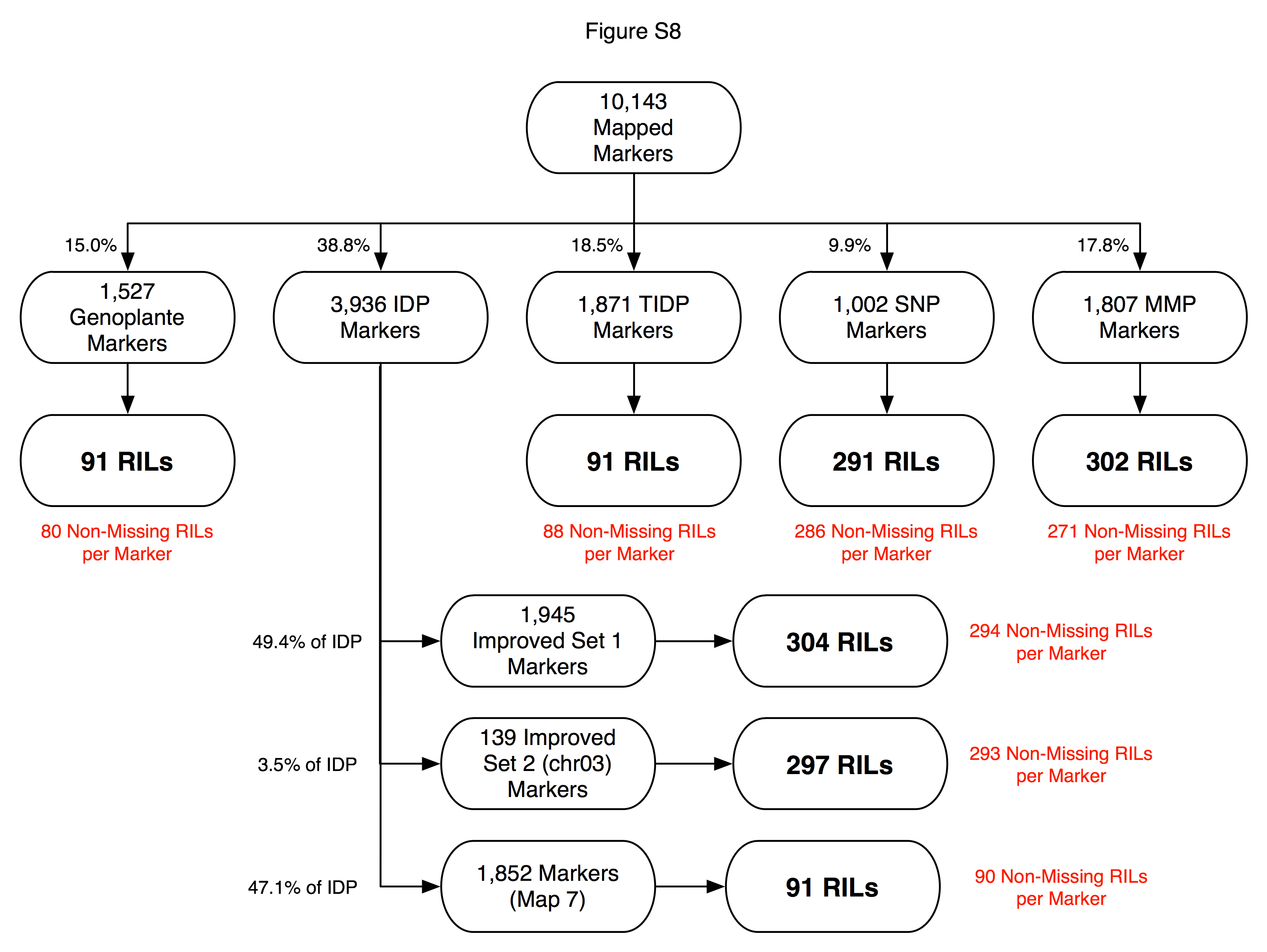

Supplement: Figure S8 — Markers used to construct the integrated genetic map. An integrated genetic map of maize was constructed based on genotyping data from 10,143 markers from multiple mapping projects (Missouri Mapping Project (MMP) (Coe et al., Plant Phys 2002, Cone et al., Plant Phys 2002), Genoplante (Falque et al., Genetics 2005), ISU-IDP/TIDP (Map 7) (unpublished), ISU SNP (Liu et al., Genetics 2010)). Some IDP markers were used to genotype additional IBM RILs as part of this study. This flowchart provides types and numbers of markers used to genotype RILs. See also Figure S7 and Tables S3 and S4. (0.56 MB TIF) [file pgen.1000733.s008.tif]
