# Supplementary material for: Mu Transposon Insertion Sites and Meiotic Recombination Events Co-Localize with Epigenetic Marks for Open Chromatin across the Maize Genome
Source: PLoS Genet. 2009 Nov 20;5(11):e1000733. doi: 10.1371/journal.pgen.1000733 (PMC2774946; doi:10.1371/journal.pgen.1000733)
Supplement: Table S1 — Novel pTIRs. (0.10 MB DOC) [file pgen.1000733.s009.doc]

**Table** **S1**. Novel pTIRs

| **Novel TIR** | **Sequences (5' - 3')** | **Closest known TIR^1^** | **Edit Distance^2^** | **No. Reads** | **No. Unique Insertions** |
| --- | --- | --- | --- | --- | --- |
| nTIR01 | TCCTTCTCTCTTCTTCTATAATGACAATTATCTC | mu3a-4 | 2 | 1,181 | 1 |
| nTIR02 | CACCTCTGTCGTGTTTTATAATGGAAAATTTCTC | mu12b | 7 | 130 | 1 |
| nTIR03 | CCCTTCCCTCTTCGTCCAGAATGACAATTATCTC | mu1a;mu2b | 2 | 132 | 1 |
| nTIR04 | CCCTCGTCTCTTCGTCCATAATGGCAAGCGCCAG | mu7a | 7 | 134 | 1 |
| nTIR05 | TCGTTCGCTCTTCGTCTATAATGGCAATTATCTC | mu8b | 2 | 138 | 1 |
| nTIR06 | CCGTCTGCTCTTTGTCTATAATGGCAATTATCTC | mu8b | 2 | 187 | 2 |
| nTIR07 | CCCTTTTCTTTTTCTTCTATAATGGCAATTATCT | mu3a-4 | 4 | 196 | 1 |
| nTIR08 | CGCATCTGCGGTGTTTCATAATCCCAAAATTCTC | mu12b | 5 | 200 | 6 |
| nTIR09 | CGCTTCTCTCTTCGTCCATAATGGCTCTTCGTCC | mu1b | 5 | 297 | 1 |
| nTIR10 | CCCTTCTTTCTTCTTTCATAATGGCAATTATCTC | mu11b | 2 | 666 | 2 |
| nTIR11 | TCTTCTCTCTTCTCTCTACTAACTATTAAGTCTC | mu3a-4 | 9 | 8,925 | 2 |
| nTIR12 | CCCTTCTCTCTTCTTCTATAACTAGGTGGATGCC | mu3a-4 | 9 | 971 | 1 |
| nTIR13 | CCCTTCTCTCTTCGTCTACAATGACAATTATCTC | mu3a-4 | 3 | 10,126 | 3 |
| nTIR14 | CCTCTCTCTCTTCGTCTATAATCGCAATTGTCTC | mu8b;mu1b;mu5b;mudr;mu3a-4;mu7a,b | 5 | 3,060 | >100 |
| nTIR15 | CCCCTTTCTTTTCTTCTATAATGGCAATTATCTT | mu3a-4 | 4 | 26,251 | 5 |
| nTIR16 | CCCTTTTCTCTTGTTCCATAATGGCAATTCTCTC | mu11b;mu3b | 3 | 1,339 | 2 |
| nTIR17 | CCCTCCTCTCTTCTTCTATAATGACAATTATCTC | mu3a-4 | 2 | 1,291 | 3 |
| nTIR18 | CCCTCCTCTCTTCGTCCATAATGACAATTATGTC | mu7a | 2 | 1,394 | 2 |
| nTIR19 | CCTTTCTCTCTTCGTCCATAATGGCAATTGTCTC | mu1b;mu1a-2b;mu5b;mu11b;mu7a | 3 | 1,980 | 3 |
| nTIR20 | CAAATCTGCCGTGTTTTATAATCCCAAAAGTCTC | mu12b | 8 | 3,470 | 2 |
| nTIR21 | CACATCCGCGGCGTTTCATAATCGCAAAAGTCTC | mu12b | 8 | 340 | 4 |

^1^ “a” and “b” designate arbitrarily defined left and right sides of each of *Mu* elements.

^2^ From nearest known pTIR
