# Supplementary material for: Mu Transposon Insertion Sites and Meiotic Recombination Events Co-Localize with Epigenetic Marks for Open Chromatin across the Maize Genome
Source: PLoS Genet. 2009 Nov 20;5(11):e1000733. doi: 10.1371/journal.pgen.1000733 (PMC2774946; doi:10.1371/journal.pgen.1000733)
Supplement: Table S2 — Frequencies of Mu insertions in different combinations of four epigenetic modifications in histone 3. (0.05 MB DOC) [file pgen.1000733.s010.doc]

Table S2. Frequencies of *Mu* insertions in different combinations of four epigenetic modifications in histone 3

| H3K4me3^1^ | H3K9ac^2^ | H3K36me3^3^ | H3K27me3^4^ | Total Length (Mb)^5^ | No. *Mu*^6^ | No. *Mu*/Mb^7^ | Notes |
| --- | --- | --- | --- | --- | --- | --- | --- |
| + | - | - | - | 6.1 | 1,971 | 245 | Only H3K4me3 |
| - | + | - | - | 14.1 | 3,230 | 205 | Only H3K9ac |
| - | - | + | - | 15.9 | 690 | 110 | Only K3K36me3 |
| - | - | - | + | 5.8 | 344 | 44 | Only H3K27me3 |
| + | + | - | - | 11.5 | 6,527 | 559 | H3K4me3&H3K9ac |
| + | - | + | - | 1.4 | 628 | 308 | H3K4me3&H3K36me3 |
| + | - | - | + | 0.9 | 107 | 126 | H3K4me3&H3K27me3 |
| - | + | + | - | 1.8 | 609 | 388 | H3K36me3&H3K9ac |
| - | + | - | + | 0.4 | 48 | 60 | H3K27me3&H3K9ac |
| - | - | + | + | 0.06 | 1 | 9 | H3K27me  3&H3K36me3 |
| + | + | + | - | 13.4 | 13,388 | 924 | H3K4me3&H3K36me3&H3K9ac |
| + | + | - | + | 1.3 | 159 | 127 | H3K4me3&H3K27me3&H3K9ac |
| + | - | + | + | 0.03 | 2 | 25 | H3K4me3&H3K27me3&H3K36me3 |
| - | + | + | + | 0.04 | 1 | 8 | H3K27me3&H3K36me3&H3K9ac |
| + | + | + | + | 0.015 | 21 | 130 | All four epi-marks |
| - | - | - | - | 10.5 | 25 | 25 | None of four epi-marks |
| Unknown | Unknown | Unknown | Unknown | 11.3 | 623 | 55 | WGS-GSS^8^ |

^1^ CHIP-seq of trimethylation of lysine 4 in histone 3; “+” represents presence; “-” represents absence

^2^ CHIP-seq of acetylation of lysine 9 in histone 3; “+” represents presence; “-” represents absence

^3^ CHIP-seq of trimethylation of lysine 36 in histone 3; “+” represents presence; “-” represents absence

^4^ CHIP-seq of trimethylation of lysine 27 in histone 3; “+” represents presence; “-” represents absence

^5^ Length (Mb) of the whole CHIP-seq regions with the corresponding histone modifications

^6^ Total number of *Mu* insertions in the CHIP-seq regions with the corresponding histone modifications

^7^ The number of *Mu* per Mb in each sequence was calculated and then those frequencies were averaged

^8^ Whole genome shotgun (WGS) - genome survey sequences (GSS) as per Fu *et al*. (2005).
