# Supplementary material for: Mu Transposon Insertion Sites and Meiotic Recombination Events Co-Localize with Epigenetic Marks for Open Chromatin across the Maize Genome
Source: PLoS Genet. 2009 Nov 20;5(11):e1000733. doi: 10.1371/journal.pgen.1000733 (PMC2774946; doi:10.1371/journal.pgen.1000733)
Supplement: Table S3 — List of 357 RILs in the integrated genetic map. (0.38 MB DOC) [file pgen.1000733.s011.doc]

**Table S3. List of 357 RILs in the integrated genetic map**

| MAP^1^ | ISU Integrated Map (N=357)^2^ | ISU Map7 (N=91)^3^ | ISU SNP (N=291)^4^ | Genoplante (N=91)^5^ | Missouri Mapping Project (MMP) (N=302)^6^ |
| --- | --- | --- | --- | --- | --- |
| M0001 | Yes | Yes | Yes | Yes | Yes |
| M0002 | Yes | No | Yes | No | No |
| M0003 | Yes | No | No | No | Yes |
| M0004 | Yes | No | Yes | No | No |
| M0005 | Yes | Yes | Yes | Yes | Yes |
| M0006 | Yes | No | Yes | No | No |
| M0007 | Yes | Yes | Yes | Yes | Yes |
| M0008 | Yes | Yes | Yes | Yes | Yes |
| M0009 | Yes | No | No | No | Yes |
| M0010 | Yes | Yes | Yes | Yes | Yes |
| M0011 | Yes | No | Yes | No | Yes |
| M0012 | Yes | Yes | Yes | Yes | Yes |
| M0013 | Yes | No | Yes | No | Yes |
| M0014 | Yes | Yes | Yes | Yes | Yes |
| M0015 | Yes | Yes | Yes | Yes | Yes |
| M0016 | Yes | Yes | Yes | Yes | Yes |
| M0017 | Yes | Yes | Yes | Yes | Yes |
| M0018 | Yes | No | No | No | Yes |
| M0019 | Yes | No | No | No | Yes |
| M0021 | Yes | Yes | Yes | Yes | Yes |
| M0022 | Yes | Yes | Yes | Yes | Yes |
| M0023 | Yes | Yes | Yes | Yes | Yes |
| M0024 | Yes | Yes | Yes | Yes | Yes |
| M0025 | Yes | Yes | Yes | Yes | Yes |
| M0026 | Yes | No | Yes | No | Yes |
| M0027 | Yes | Yes | Yes | Yes | Yes |
| M0028 | Yes | Yes | Yes | Yes | Yes |
| M0029 | Yes | Yes | Yes | Yes | Yes |
| M0030 | Yes | Yes | Yes | Yes | Yes |
| M0031 | Yes | Yes | Yes | Yes | Yes |
| M0032 | Yes | Yes | Yes | Yes | Yes |
| M0033 | Yes | Yes | Yes | Yes | Yes |
| M0034 | Yes | Yes | Yes | Yes | Yes |
| M0035 | Yes | Yes | Yes | Yes | Yes |
| M0036 | Yes | No | No | No | Yes |
| M0038 | Yes | No | No | No | Yes |
| M0039 | Yes | Yes | Yes | Yes | Yes |
| M0040 | Yes | Yes | Yes | Yes | Yes |
| M0041 | Yes | No | No | No | Yes |
| M0042 | Yes | No | No | No | Yes |
| M0043 | Yes | Yes | Yes | Yes | Yes |
| M0044 | Yes | No | Yes | No | Yes |
| M0045 | Yes | Yes | Yes | Yes | Yes |
| M0046 | Yes | Yes | Yes | Yes | Yes |
| M0047 | Yes | No | Yes | No | Yes |
| M0048 | Yes | Yes | Yes | Yes | Yes |
| M0051 | Yes | Yes | Yes | Yes | Yes |
| M0052 | Yes | Yes | Yes | Yes | Yes |
| M0053 | Yes | No | Yes | No | No |
| M0054 | Yes | Yes | Yes | Yes | Yes |
| M0055 | Yes | Yes | Yes | Yes | Yes |
| M0056 | Yes | No | Yes | No | Yes |
| M0057 | Yes | Yes | Yes | Yes | Yes |
| M0058 | Yes | Yes | Yes | Yes | Yes |
| M0059 | Yes | No | Yes | No | Yes |
| M0060 | Yes | Yes | Yes | Yes | Yes |
| M0061 | Yes | Yes | Yes | Yes | Yes |
| M0062 | Yes | No | No | No | Yes |
| M0063 | Yes | Yes | No | Yes | Yes |
| M0064 | Yes | No | Yes | No | No |
| M0065 | Yes | No | Yes | No | No |
| M0066 | Yes | Yes | Yes | Yes | Yes |
| M0067 | Yes | Yes | Yes | Yes | Yes |
| M0068 | Yes | No | No | No | Yes |
| M0069 | Yes | No | Yes | No | No |
| M0070 | Yes | No | Yes | No | No |
| M0071 | Yes | No | Yes | No | Yes |
| M0073 | Yes | No | No | No | No |
| M0074 | Yes | Yes | No | Yes | Yes |
| M0075 | Yes | Yes | Yes | Yes | Yes |
| M0076 | Yes | Yes | Yes | Yes | Yes |
| M0077 | Yes | Yes | Yes | Yes | Yes |
| M0078 | Yes | No | Yes | No | No |
| M0079 | Yes | Yes | Yes | Yes | Yes |
| M0080 | Yes | No | Yes | No | Yes |
| M0081 | Yes | No | Yes | No | Yes |
| M0082 | Yes | No | No | No | Yes |
| M0083 | Yes | No | Yes | No | Yes |
| M0084 | Yes | No | Yes | No | No |
| M0085 | Yes | No | Yes | No | Yes |
| M0086 | Yes | No | Yes | No | Yes |
| M0087 | Yes | No | No | No | Yes |
| M0088 | Yes | No | Yes | No | Yes |
| M0090 | Yes | No | Yes | No | Yes |
| M0091 | Yes | No | Yes | No | Yes |
| M0092 | Yes | No | Yes | No | Yes |
| M0093 | Yes | No | Yes | No | Yes |
| M0095 | Yes | No | Yes | No | Yes |
| M0096 | Yes | No | Yes | No | Yes |
| M0097 | Yes | No | Yes | No | Yes |
| M0098 | Yes | No | Yes | No | Yes |
| M0099 | Yes | No | Yes | No | Yes |
| M0100 | Yes | No | Yes | No | No |
| M0101 | Yes | No | Yes | No | Yes |
| M0102 | Yes | No | Yes | No | Yes |
| M0103 | Yes | No | Yes | No | No |
| M0104 | Yes | No | Yes | No | No |
| M0105 | Yes | No | Yes | No | Yes |
| M0106 | Yes | No | Yes | No | Yes |
| M0107 | Yes | No | Yes | No | No |
| M0109 | Yes | No | Yes | No | Yes |
| M0110 | Yes | No | Yes | No | Yes |
| M0111 | Yes | No | No | No | Yes |
| M0113 | Yes | No | Yes | No | Yes |
| M0114 | Yes | No | Yes | No | Yes |
| M0115 | Yes | No | Yes | No | Yes |
| M0116 | Yes | No | Yes | No | Yes |
| M0117 | Yes | No | Yes | No | No |
| M0118 | Yes | No | Yes | No | Yes |
| M0119 | Yes | No | No | No | Yes |
| M0120 | Yes | No | Yes | No | Yes |
| M0121 | Yes | No | Yes | No | Yes |
| M0122 | Yes | No | Yes | No | No |
| M0123 | Yes | No | Yes | No | Yes |
| M0124 | Yes | No | Yes | No | Yes |
| M0125 | Yes | No | Yes | No | Yes |
| M0126 | Yes | No | Yes | No | Yes |
| M0127 | Yes | No | Yes | No | Yes |
| M0128 | Yes | No | No | No | Yes |
| M0129 | Yes | No | Yes | No | Yes |
| M0130 | Yes | No | Yes | No | Yes |
| M0131 | Yes | No | Yes | No | Yes |
| M0132 | Yes | No | Yes | No | Yes |
| M0133 | Yes | No | Yes | No | Yes |
| M0134 | Yes | No | No | No | Yes |
| M0136 | Yes | No | No | No | Yes |
| M0138 | Yes | No | Yes | No | Yes |
| M0141 | Yes | No | Yes | No | Yes |
| M0142 | Yes | No | Yes | No | Yes |
| M0143 | Yes | No | Yes | No | Yes |
| M0144 | Yes | No | Yes | No | No |
| M0145 | Yes | No | Yes | No | Yes |
| M0146 | Yes | No | No | No | Yes |
| M0147 | Yes | No | Yes | No | Yes |
| M0148 | Yes | No | No | No | Yes |
| M0149 | Yes | No | Yes | No | No |
| M0150 | Yes | No | Yes | No | Yes |
| M0151 | Yes | No | Yes | No | Yes |
| M0152 | Yes | No | Yes | No | Yes |
| M0153 | Yes | No | No | No | Yes |
| M0154 | Yes | No | Yes | No | Yes |
| M0155 | Yes | No | Yes | No | No |
| M0156 | Yes | No | Yes | No | Yes |
| M0157 | Yes | No | Yes | No | Yes |
| M0159 | Yes | No | No | No | Yes |
| M0160 | Yes | No | Yes | No | Yes |
| M0160A | Yes | No | Yes | No | No |
| M0161 | Yes | No | Yes | No | Yes |
| M0162 | Yes | No | Yes | No | Yes |
| M0162A | Yes | No | Yes | No | No |
| M0163 | Yes | No | Yes | No | Yes |
| M0164 | Yes | No | No | No | Yes |
| M0165 | Yes | No | Yes | No | Yes |
| M0166 | Yes | No | Yes | No | No |
| M0167 | Yes | No | Yes | No | Yes |
| M0168 | Yes | No | Yes | No | Yes |
| M0169 | Yes | No | Yes | No | Yes |
| M0170 | Yes | No | No | No | Yes |
| M0171 | Yes | No | No | No | Yes |
| M0172 | Yes | No | No | No | Yes |
| M0173 | Yes | No | Yes | No | No |
| M0174 | Yes | No | Yes | No | Yes |
| M0175 | Yes | No | No | No | Yes |
| M0176 | Yes | No | Yes | No | Yes |
| M0177 | Yes | No | Yes | No | Yes |
| M0178 | Yes | No | Yes | No | Yes |
| M0179 | Yes | No | Yes | No | No |
| M0180 | Yes | No | Yes | No | Yes |
| M0181 | Yes | No | Yes | No | Yes |
| M0182 | Yes | No | No | No | Yes |
| M0183 | Yes | No | No | No | Yes |
| M0184 | Yes | No | No | No | Yes |
| M0185 | Yes | No | Yes | No | Yes |
| M0186 | Yes | No | No | No | Yes |
| M0187 | Yes | No | Yes | No | Yes |
| M0188 | Yes | No | Yes | No | Yes |
| M0189 | Yes | No | Yes | No | Yes |
| M0190 | Yes | No | No | No | Yes |
| M0191 | Yes | No | Yes | No | Yes |
| M0192 | Yes | No | Yes | No | Yes |
| M0194 | Yes | No | Yes | No | Yes |
| M0195 | Yes | No | Yes | No | Yes |
| M0196 | Yes | No | Yes | No | Yes |
| M0197 | Yes | No | Yes | No | Yes |
| M0198 | Yes | No | Yes | No | Yes |
| M0199 | Yes | No | Yes | No | Yes |
| M0200 | Yes | No | Yes | No | Yes |
| M0201 | Yes | No | Yes | No | Yes |
| M0202 | Yes | No | No | No | Yes |
| M0203 | Yes | No | Yes | No | No |
| M0204 | Yes | No | Yes | No | Yes |
| M0205 | Yes | No | Yes | No | Yes |
| M0206 | Yes | No | Yes | No | Yes |
| M0208 | Yes | No | Yes | No | Yes |
| M0209 | Yes | No | Yes | No | Yes |
| M0210 | Yes | No | Yes | No | Yes |
| M0212 | Yes | No | Yes | No | No |
| M0213 | Yes | No | Yes | No | Yes |
| M0214 | Yes | No | Yes | No | Yes |
| M0215 | Yes | No | Yes | No | Yes |
| M0216 | Yes | No | Yes | No | Yes |
| M0217 | Yes | No | Yes | No | No |
| M0218 | Yes | No | Yes | No | Yes |
| M0219 | Yes | No | Yes | No | Yes |
| M0220 | Yes | No | Yes | No | Yes |
| M0221 | Yes | No | No | No | No |
| M0222 | Yes | No | Yes | No | Yes |
| M0223 | Yes | No | Yes | No | Yes |
| M0224 | Yes | No | No | No | Yes |
| M0225 | Yes | No | Yes | No | Yes |
| M0228 | Yes | No | Yes | No | Yes |
| M0229 | Yes | No | Yes | No | Yes |
| M0230 | Yes | No | Yes | No | Yes |
| M0232 | Yes | No | Yes | No | Yes |
| M0233 | Yes | No | Yes | No | Yes |
| M0234 | Yes | No | Yes | No | No |
| M0235 | Yes | No | Yes | No | No |
| M0236 | Yes | No | Yes | No | Yes |
| M0237 | Yes | No | No | No | Yes |
| M0238 | Yes | No | Yes | No | Yes |
| M0239 | Yes | No | Yes | No | No |
| M0240 | Yes | No | Yes | No | Yes |
| M0241 | Yes | No | Yes | No | Yes |
| M0244 | Yes | No | Yes | No | Yes |
| M0245 | Yes | No | Yes | No | No |
| M0246 | Yes | No | No | No | Yes |
| M0248 | Yes | No | Yes | No | Yes |
| M0249 | Yes | No | Yes | No | No |
| M0250 | Yes | No | No | No | Yes |
| M0251 | Yes | No | Yes | No | No |
| M0252 | Yes | No | Yes | No | No |
| M0253 | Yes | No | No | No | Yes |
| M0254 | Yes | No | Yes | No | No |
| M0255 | Yes | No | No | No | Yes |
| M0256 | Yes | No | Yes | No | Yes |
| M0257 | Yes | No | Yes | No | No |
| M0258 | Yes | No | Yes | No | Yes |
| M0259 | Yes | No | Yes | No | No |
| M0260 | Yes | No | Yes | No | No |
| M0261 | Yes | No | No | No | Yes |
| M0262 | Yes | Yes | Yes | Yes | Yes |
| M0263 | Yes | No | Yes | No | Yes |
| M0264 | Yes | Yes | Yes | Yes | Yes |
| M0265 | Yes | Yes | Yes | Yes | Yes |
| M0266 | Yes | Yes | Yes | Yes | Yes |
| M0267 | Yes | Yes | Yes | Yes | Yes |
| M0268 | Yes | No | No | No | Yes |
| M0269 | Yes | Yes | Yes | Yes | Yes |
| M0270 | Yes | No | Yes | No | Yes |
| M0271 | Yes | No | Yes | No | Yes |
| M0272 | Yes | Yes | Yes | Yes | Yes |
| M0273 | Yes | No | Yes | No | No |
| M0274 | Yes | No | Yes | No | Yes |
| M0275 | Yes | Yes | Yes | Yes | Yes |
| M0276 | Yes | Yes | Yes | Yes | Yes |
| M0277 | Yes | No | No | No | Yes |
| M0278 | Yes | No | No | No | Yes |
| M0279 | Yes | No | Yes | No | Yes |
| M0280 | Yes | No | Yes | No | Yes |
| M0281 | Yes | Yes | Yes | Yes | Yes |
| M0282 | Yes | No | Yes | No | Yes |
| M0283 | Yes | No | Yes | No | Yes |
| M0284 | Yes | Yes | Yes | Yes | Yes |
| M0285 | Yes | No | Yes | No | Yes |
| M0286 | Yes | No | Yes | No | Yes |
| M0287 | Yes | Yes | Yes | Yes | Yes |
| M0288 | Yes | Yes | Yes | Yes | Yes |
| M0289 | Yes | No | Yes | No | Yes |
| M0290 | Yes | No | Yes | No | No |
| M0291 | Yes | No | Yes | No | No |
| M0292 | Yes | No | Yes | No | Yes |
| M0293 | Yes | No | Yes | No | No |
| M0294 | Yes | No | Yes | No | No |
| M0295 | Yes | No | Yes | No | Yes |
| M0296 | Yes | Yes | Yes | Yes | Yes |
| M0297 | Yes | Yes | Yes | Yes | Yes |
| M0298 | Yes | Yes | Yes | Yes | Yes |
| M0300 | Yes | No | Yes | No | Yes |
| M0301 | Yes | No | No | No | Yes |
| M0302 | Yes | No | No | No | Yes |
| M0303 | Yes | No | Yes | No | Yes |
| M0304 | Yes | No | Yes | No | Yes |
| M0305 | Yes | No | Yes | No | Yes |
| M0306 | Yes | No | Yes | No | No |
| M0307 | Yes | No | Yes | No | Yes |
| M0308 | Yes | No | Yes | No | Yes |
| M0309 | Yes | Yes | Yes | Yes | Yes |
| M0310 | Yes | Yes | Yes | Yes | Yes |
| M0311 | Yes | Yes | Yes | Yes | Yes |
| M0312 | Yes | No | Yes | No | No |
| M0313 | Yes | No | Yes | No | Yes |
| M0314 | Yes | No | No | No | Yes |
| M0315 | Yes | Yes | No | Yes | Yes |
| M0316 | Yes | No | No | No | Yes |
| M0317 | Yes | Yes | Yes | Yes | Yes |
| M0318 | Yes | No | Yes | No | Yes |
| M0319 | Yes | No | No | No | Yes |
| M0320 | Yes | No | Yes | No | Yes |
| M0321 | Yes | Yes | Yes | Yes | Yes |
| M0322 | Yes | Yes | Yes | Yes | Yes |
| M0323 | Yes | Yes | Yes | Yes | Yes |
| M0324 | Yes | No | Yes | No | No |
| M0325 | Yes | Yes | Yes | Yes | Yes |
| M0326 | Yes | Yes | Yes | Yes | Yes |
| M0327 | Yes | No | Yes | No | Yes |
| M0328 | Yes | Yes | Yes | Yes | Yes |
| M0329 | Yes | No | No | No | Yes |
| M0331 | Yes | No | Yes | No | Yes |
| M0332 | Yes | No | No | No | Yes |
| M0334 | Yes | No | Yes | No | Yes |
| M0335 | Yes | No | Yes | No | Yes |
| M0336 | Yes | No | No | No | Yes |
| M0337 | Yes | Yes | Yes | Yes | Yes |
| M0338 | Yes | No | Yes | No | Yes |
| M0339 | Yes | No | Yes | No | No |
| M0340 | Yes | No | No | No | Yes |
| M0341 | Yes | Yes | Yes | Yes | Yes |
| M0342 | Yes | No | Yes | No | Yes |
| M0342 (PlateC/G9) | Yes | No | No | No | No |
| M0344 | Yes | Yes | Yes | Yes | Yes |
| M0345 | Yes | Yes | Yes | Yes | Yes |
| M0346 | Yes | Yes | No | Yes | Yes |
| M0347 | Yes | No | No | No | Yes |
| M0348 | Yes | No | Yes | No | No |
| M0349 | Yes | No | No | No | Yes |
| M0350 | Yes | No | Yes | No | No |
| M0351 | Yes | No | Yes | No | Yes |
| M0352 | Yes | Yes | Yes | Yes | Yes |
| M0353 | Yes | No | Yes | No | Yes |
| M0354 | Yes | Yes | Yes | Yes | Yes |
| M0355 | Yes | Yes | Yes | Yes | Yes |
| M0356 | Yes | No | No | No | Yes |
| M0357 | Yes | Yes | Yes | Yes | Yes |
| M0358 | Yes | No | Yes | No | Yes |
| M0360 | Yes | Yes | Yes | Yes | Yes |
| M0361 | Yes | No | No | No | Yes |
| M0362 | Yes | No | Yes | No | Yes |
| M0364 | Yes | Yes | Yes | Yes | Yes |
| M0365 | Yes | Yes | Yes | Yes | Yes |
| M0366 | Yes | No | Yes | No | Yes |
| M0367 | Yes | No | No | No | Yes |
| M0368 | Yes | Yes | Yes | Yes | Yes |
| M0369 | Yes | Yes | Yes | Yes | Yes |
| M0370 | Yes | No | Yes | No | No |
| M0372 | Yes | No | No | No | Yes |
| M0373 | Yes | No | No | No | Yes |
| M0374 | Yes | No | No | No | Yes |
| M0375 | Yes | No | Yes | No | No |
| M0376 | Yes | No | Yes | No | Yes |
| M0377 | Yes | No | Yes | No | No |
| M0378 | Yes | Yes | Yes | Yes | Yes |
| M0379 | Yes | Yes | Yes | Yes | Yes |
| M0380 | Yes | No | Yes | No | Yes |
| M0381 | Yes | No | Yes | No | Yes |
| M0382 | Yes | Yes | Yes | Yes | Yes |
| M0383 | Yes | No | Yes | No | Yes |
| M0384 | Yes | Yes | Yes | Yes | Yes |

^1^ Yes: Used in the indicated map; No: not used in the indicated map

^2^ This study

^3^ Unpublished

^4^ Liu *et al*., Genetics, 2010

^5^ Falque *et al*., Genetics 2005

^6^ Coe *et al*., Plant Phys 2002; Cone *et al*., Plant Phys 2002
